# Callose-mediated resistance to pathogenic intruders in plant defense-related papillae

**DOI:** 10.3389/fpls.2014.00168

**Published:** 2014-04-28

**Authors:** Christian A. Voigt

**Affiliations:** Phytopathology and Biochemistry, Biocenter Klein Flottbek, University of HamburgHamburg, Germany

**Keywords:** callose, defense response, innate immunity, penetration resistance, plant-pathogen interaction

## Abstract

Plants are exposed to a wide range of potential pathogens, which derive from diverse phyla. Therefore, plants have developed successful defense mechanisms during co-evolution with different pathogens. Besides many specialized defense mechanisms, the plant cell wall represents a first line of defense. It is actively reinforced through the deposition of cell wall appositions, so-called papillae, at sites of interaction with intruding microbial pathogens. The papilla is a complex structure that is formed between the plasma membrane and the inside of the plant cell wall. Even though the specific biochemical composition of papillae can vary between different plant species, some classes of compounds are commonly found which include phenolics, reactive oxygen species, cell wall proteins, and cell wall polymers. Among these polymers, the (1,3)-*β*-glucan callose is one of the most abundant and ubiquitous components. Whereas the function of most compounds could be directly linked with cell wall reinforcement or an anti-microbial effect, the role of callose has remained unclear. An evaluation of recent studies revealed that the timing of the different papilla-forming transport processes is a key factor for successful plant defense.

## INTRODUCTION

During plant-pathogen co-evolution, plants have evolved a range of defense mechanisms to prevent ingress and colonization by potential pathogens, which derive from diverse phyla and include fungi, oomycetes, animals, bacteria, and viruses. Successful plant defense depends on an early and rapid perception of the invading pathogen and subsequent induction and mobilization of biochemical and structural defense-related mechanisms. In most cases, plant defense to pathogens is successful and infection is the exception, which reflects the general effectiveness of the plant’s immune response (Deverall, 1977; Smith, 1978; Bailey, 1983; Thordal-Christensen, 2003).

The perception of pathogens and the induced plant defense response generally follows two branches of the active immune system. R proteins, which are encoded by plant disease resistance (*R*) genes, control plant defense (Flor, 1971) by recognizing the presence of corresponding avirulence (avr) proteins or effectors deriving from the pathogen. This can occur through direct binding of the avr protein or effector, the binding of an effector-modified target, or recognition of an effector/target complex (Dodds and Rathjen, 2010). In extension to these binding possibilities, a current model suggests that R proteins can guard key cellular hubs, which might be common targets of effectors from different pathogen origin (Mukhtar et al., 2011). Even though most of the R proteins identified contain a nucleotide binding site (NBS) and leucine-rich repeats (LRRs), and are localized intracellularly, a growing number of R proteins has been discovered that contain membrane anchorage motifs, e.g., a myristoylation motif, and an extracellular LRR but lack a NBS (Dangl and Jones, 2001; Martin et al., 2003; Glowacki et al., 2011). Plant NBS-LRR- or atypical R protein-mediated pathogen resistance is only effective against obligate biotrophic or hemi-biotrophic pathogens that require living host tissue for propagation. However, plant NBS-LRR- or atypical R protein-mediated pathogen resistance is not effective against necrotrophic pathogens that macerate and degrade host tissue during colonization (Glazebrook, 2005). In the second arm of the active immune system, transmembrane pattern recognition receptors (PRR) that perceive microbe- or pathogen molecular patterns (MAMPs; Ausubel, 2005) respond to intruding pathogens (Jones and Takemoto, 2004; Nürnberger et al., 2004). MAMPs are characterized by their general occurrence in all members of a pathogen class and their often requirement for pathogen viability. Prominent examples for MAMPs are flagellin from bacterial pathogens and chitin from fungal pathogens, for which the respective PRR have been identified and characterized (Chinchilla et al., 2006; Kaku et al., 2006; Chinchilla et al., 2007; Miya et al., 2007). The widely accepted four phased Zig-Zag model from Jones and Dangl (2006) represents how these two branches of the plant immune system interact.

The plant immune system triggers a variety of defense mechanisms and include a hypersensitive response (HR) for rapid collapse of attacked host cells (Coll et al., 2011), production of anti-microbial phytoalexins (Bednarek and Osbourn, 2009), biosynthesis of enzymes, which can decompose pathogen cell walls (Mauch et al., 1988), and plant cell wall modifications; notably the deposition of papillae, which are enriched with the (1,3)-*β*-glucan cell wall polymer callose (Aist, 1976). These cell wall thickenings are formed at sites of microbial attack and are thought to act as a physical barrier to slow pathogen invasion (Stone and Clarke, 1992). Compared with many plant defense responses that can be specific to a phylum or even a species, the formation of callose-rich papillae can be regarded as a ubiquitous response because it appears to be induced in essentially all plants following pathogen challenge.

## THE ROLE OF CALLOSE IN PATHOGEN-INDUCED PAPILLAE

The formation of papillae is one of the earliest observed plant defense responses that has been analyzed on a cellular level for over 150 years. deBary (1863) discovered papillae at sites of fungal penetration, and Mangin (1895) reported that callose commonly occurs in papillae. Since then, chemical analyses have identified additional chemical components, which comprise phenolic compounds and lignin, an additional cell wall polymer to callose, reactive oxygen species (ROS), and cell wall proteins like peroxidases and anti-microbial thionins (Aist and Williams, 1971; Mercer et al., 1974; McLusky et al., 1999; Mims et al., 2000). Whereas the role of callose in papillae remained unclear, a function in defense could be attributed to most of the other components. For instance, hydrogen peroxide is a ROS that accumulates in forming papillae and can be used by peroxidases to promote cross-linking of proteins and phenolics to reinforce cell wall appositions (Thordal-Christensen et al., 1997; Brown et al., 1998).

In general, papillae formation is an early defense response and can contribute to the plant’s innate immunity (Jones and Dangl, 2006; Schwessinger and Ronald, 2012). By slowing pathogen invasion in the attacked tissue, papillae formation can gain time for an induction of additional defense responses that may require gene activation and expression (Lamb and Dixon, 1997; Brown et al., 1998; Boller and Felix, 2009). However, the extent to which papillae and the deposited callose would contribute to the plant’s innate immunity and penetration resistance has been subject to an ongoing discussion.

Callose-rich papillae were not only found in cases of successful resistance but also at sites of pathogen penetration (Aist, 1976). In this regard, the proposed function of callose in strengthening cell wall appositions and contributing to penetration resistance was further challenged by studies using *Arabidopsis* (*Arabidopsis thaliana*) mutants. Disruption mutants that lack the stress-induced callose synthase PMR4 [POWDERY MILDEW RESISTANT 4; also known as GSL5 (GLUCAN SYNTHASE-LIKE 5)] and do not deposit callose at sites of attempted fungal penetration, showed an unexpected, increased resistance to powdery mildew species (Jacobs et al., 2003; Nishimura et al., 2003). This result revealed that in *Arabidopsis* wild-type leaves, callose levels at penetration sites do not contribute to penetration resistance to adapted powdery mildews. However, callose deposition is required to maintain the high penetration resistance to the non-adapted powdery mildew *Blumeria graminis* f.sp. *hordei*, which was challenged in *pmr4* mutants (Jacobs et al., 2003; Ellinger et al., 2013). Additional double-mutant and microarray analyses revealed that the hyperactivated salicylic acid (SA) pathway caused the high resistance to adapted powdery mildews in *pmr4* mutants (Nishimura et al., 2003). The SA-dependent resistance of *pmr4* mutants, however, might not be directly related to missing callose because the penetration success of an adapted powdery mildew was not different between wild-type and *pmr4* mutant at early stages of infection (Consonni et al., 2010; Ellinger et al., 2013).

Contrary results about an active role of callose in forming papillae derived from studies using *mlo* (*MILDEW RESISTANCE LOCUS O*) disruption mutants. In *Arabidopsis*, the observed *mlo2*-conditioned penetration resistance to powdery mildew did not require PMR4-dependent callose formation (Consonni et al., 2010) whereas *mlo*-resistant barley (*Hordeum vulgare*) coleptiles seemed to be dependent on papillae containing callose to maintain their penetration resistance to powdery mildew (Bayles et al., 1990). However, it has to be considered that the results in barley were based on a treatment with a callose synthase inhibitor that is not specific to stress-induced callose biosynthesis. Therefore, inhibition of additional callose synthases might have contributed to increased penetration susceptibility in this experiment. In our recent study, we could directly confirm that callose deposition in papillae can have an active role in penetration resistance also in *Arabidopsis*. The overexpression of *PMR4* caused an elevated early callose deposition at sites of attempted fungal penetration, which provided complete penetration resistance to the adapted powdery mildew *Golovinomyces cichoracearum* and the non-adapted powdery mildew *B. graminis* (Ellinger et al., 2013). This example reveals that timing and rapid transportation in papillae formation is important to slow or even stop pathogen invasion.

The extent to which overexpression of *PMR4* could be applied in crops to induce callose-mediated pathogen resistance is currently under investigation. First results from increased penetration resistance to the virulent powdery mildew *B. graminis* in barley leaves after transient *PMR4* overexpression (Blümke et al., 2013) prompted us to generate stable *PMR4* expression lines in barley, wheat (*Triticum aestivum*), and the model grass *Brachypodium distachyon*. To test whether induced penetration resistance would be dependent on overexpression of the callose synthase gene *PMR4* from *Arabidopsis*, callose synthase genes from the respective plant of interest could be used for overexpression. In this regard, new breeding strategies, like non-GMO-considered TAL effector nuclease mutagenesis (Wendt et al., 2013) would open new possibilities in applying this type of induced resistance in crops, e.g., by site-directed editing of the promoter region for constitutive expression of the gene of interest.

## REGULATION OF TRANSPORT PROCESSES AT THE FORMING PAPILLAE

The spatial confinement of papillae to the paramural space between the cell wall and the plasma membrane at sites of attempted pathogen penetration suggests a site-directed transport of papilla components and cell wall-synthesizing enzymes, which would imply an induction and regulation of cell polarization processes (Schmelzer, 2002; Koh et al., 2005). The rearrangement of the cytoskeleton is an important factor in these processes. Actin filaments might be especially involved in the delivery of vesicles and transportation of organelles, like the Golgi and the nucleus, to the infection site and the forming papilla. This was supported in experiments where actin formation and rearrangements were inhibited, which resulted in increased fungal penetration (Kobayashi et al., 1997; Yun et al., 2003). Also *mlo*-mediated penetration resistance to powdery mildew was shown to be dependent on active actin reorganization (Opalski et al., 2005; Miklis et al., 2007). However, the MLO protein itself accumulated at the forming papillae at penetration sites in absence of an intact actin cytoskeleton (Bhat et al., 2005). This suggests that also actin-independent mechanisms for protein recruitment to infection sites may exist. MLO was also shown to negatively regulate penetration resistance to powdery mildew because in different plant species, like barley, tomato (*Solanum lycopersicum*), and *Arabidopsis*, mutation or disruption of the *MLO* locus conferred increased resistance (Jørgensen, 1992; Piffanelli et al., 2004; Consonni et al., 2006; Bai et al., 2008; Consonni et al., 2010). A putative interaction of MLO and ROR2 (REQUIRED FOR *mlo* RESISTANCE 2) in barley and PEN1 (PENETRATION 1) in *Arabidopsis* (Schulze-Lefert, 2004; Bhat et al., 2005; Panstruga, 2005) could link MLO function with the regulation of transport processes to papillae. However, genetic data suggest that MLO and ROR2/PEN1 function independently. Therefore, observed effects are likely additive and would not support a direct functional link on a molecular level.

PEN1 and ROR2 are functionally homologous members of the syntaxin family (Collins et al., 2003). Similar to target SNAP (SOLUBLE NSF ATTACHEMENT PROTEIN) receptors (tSNARE), syntaxins form ternary SNARE complexes with corresponding VAMPs (VESICLE ASSOCIATED MEMBRANE PROTEIN), which reflects their direct involvement in vesicle fusion processes. PEN1 and ROR2 accumulation at the plasma membrane at sites of attempted fungal penetration allowed a further specification of their function in targeting vesicle trafficking to the forming papilla (Assaad et al., 2004; Bhat et al., 2005; Underwood and Somerville, 2008). This possible function of PEN1 was supported by findings in *Arabidopsis pen1* disruption mutants where papillae formation was reduced at early time-points of powdery mildew infection due to a delay in papilla deposition (Assaad et al., 2004). However, *pen1* disruption did not change the general morphology of papillae, which might indicate an involvement of one or more additional syntaxins that could substitute PEN1 during papilla formation. In contrast to PEN1, PEN2 was found to localize to peroxisomes. These organelles accumulate at sites of attempted fungal penetration where they are thought to deliver compounds with a potential antifungal activity to papillae. Because *PEN2* encodes a glycosyl hydrolase, it might directly participate in compound formation (Lipka et al., 2005; Bednarek et al., 2009). Similar to PEN1, the ATP-binding cassette (ABC) transporter PEN3 localized to the plasma membrane in unchallenged *Arabidopsis* leaves and was transported to the site of papilla formation after pathogen attack where it strongly accumulated (Stein et al., 2006; Underwood and Somerville, 2008). Interestingly, *pen3* disruption mutants revealed a higher resistance to biotrophic and hemibiotrophic pathogens, which was associated with an upregulation of SA biosynthesis or signaling and induced HR-like cell death (Kobae et al., 2006; Stein et al., 2006). Based on the transporter function and the observed phenotypes of *PEN3* mutants, it has been proposed that PEN3 might participate in the export of anti-microbial compounds, which could derive from PEN2-dependent processing in peroxisomes. For its recruitment and focal accumulation at sites of attempted penetration, PEN3 required functional actin filaments but not microtubules, secretory trafficking or protein biosynthesis. Hence, an unknown trafficking pathway might be involved in translocation of existing PEN3 (Underwood and Somerville, 2013).

Regarding one of the most prominent components of the forming papilla, the localized deposition of the (1,3)-*β*-glucan callose in response to pathogen attack suggests a precise timing of preceding transport processes (Zimmerli et al., 2004; Koh et al., 2005; Nielsen et al., 2012; Ellinger et al., 2013). Our recent results of the overexpression of the GFP-tagged callose synthase PMR4 in *Arabidopsis* suggested that this enzyme was released from vesicle-like bodies and reintegrated into the plasma membrane at sites of attempted penetration where callose deposition started (Ellinger et al., 2013). Therefore, recruitment from the plasma membrane and transport in vesicle-like bodies to the site of attempted penetration could be anticipated after fungal infection. This would also explain why application of brefeldin A, which is a known inhibitor of vesicle transport (Sciaky et al., 1997), prevented callose accumulation at forming papillae (Nielsen et al., 2012). However, a direct proof of PMR4 transportation to sites of attempted pathogen penetration in vesicles is still missing.

In the current discussion about vesicles that are required for transportation and delivery of defense components to the forming papilla, growing evidence of an involvement of multi-vesicular bodies (MVBs) has been provided. MVBs mediate exocytosis that would facilitate a delivery of vesicles and their content to the cell exterior, namely the forming papilla in the paramural space between the plasma membrane and the cell wall. In barley, MVBs contained the ADP-ribosylation factor (ARF) GTPase ARFA1b/1c that was required for callose deposition in papillae and penetration resistance to the powdery mildew *B. graminis*; but absence of this GTPase did not prevent basic papillae formation (Böhlenius et al., 2010). The importance of a timely and coordinated delivery of components required for proper papillae formation was further supported in experiments with *Arabidopsis* where mutation of the ARF-GTP exchange factor GNOM resulted in a 30 min delay of callose deposition in papillae (Nielsen et al., 2012).

## A NEW DIRECTION OF PAPILLA EXPENSION

The currently discussed and presented models of papillae formation favor a transportation and delivery of papilla components by vesicles, which are controlled by a complex, underlying network of regulatory mechanisms. This would ensure a rapid and coordinated assembly of this pathogen-induced cell wall structure to support penetration resistance.

**FIGURE 1:**
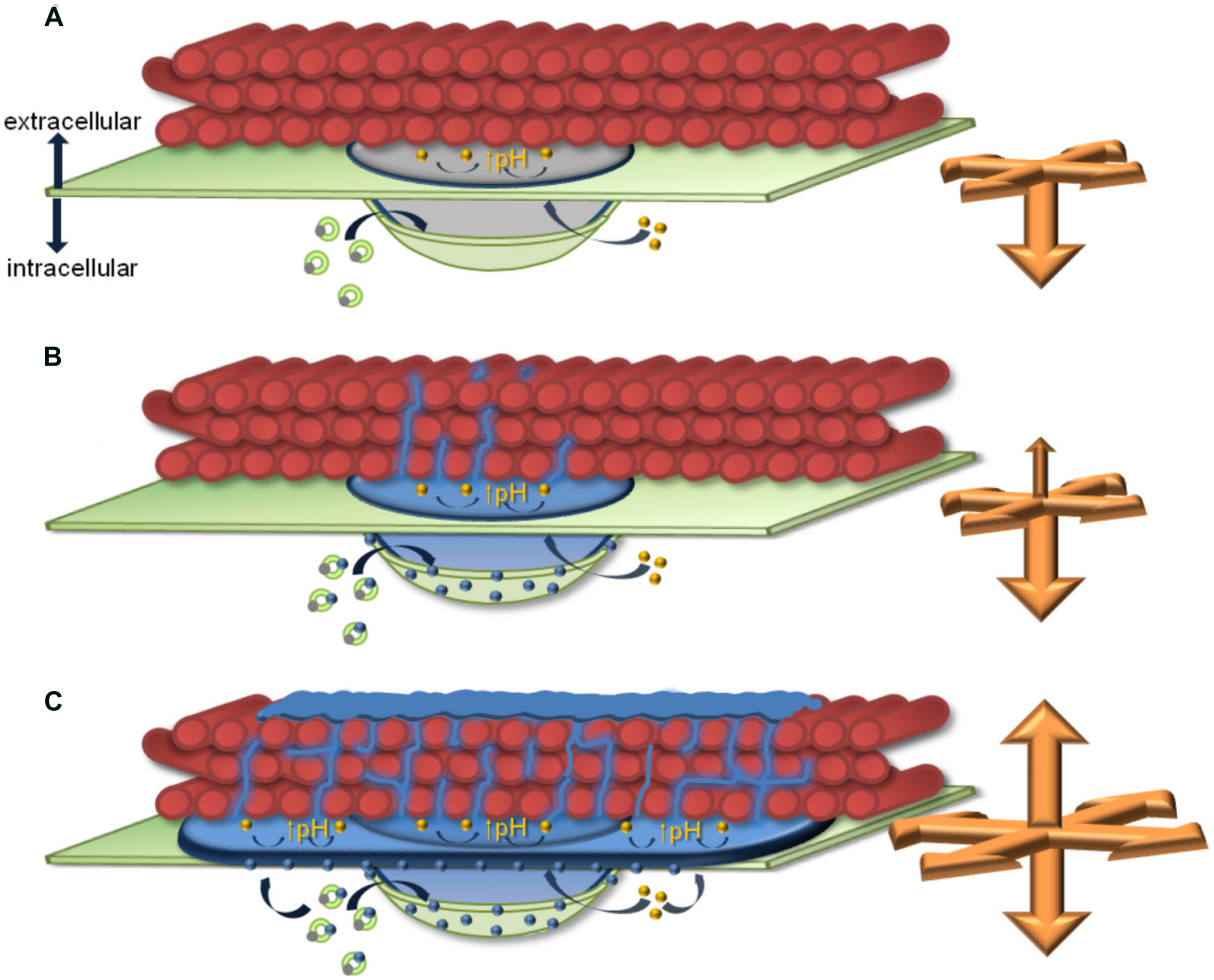
**Model of callosic papilla expansion at infection sites.** The presented model highlights similarities and differences of callosic papilla expansion and callose/cellulose polymer network formation in *Arabidopsis* epidermal leaf cells at sites of attempted powder-mildew infection in **(A)** the *pmr4* disruption mutant without pathogen-induced callose deposition in the papilla, **(B)** wild-type, and **(C)** the penetration-resistant *PMR4* overexpression line. Green circles represent possible multi-vesicular bodies (MVBs) involved in the delivery of non-callosic papilla matrix and/or papilla-forming enzymes (gray dots) and the callose synthase PMR4 (blue dots) to the forming papilla. Yellow dots inside the papilla matrix indicate a putative involvement of vesicles/vesicle-like bodies in regulating the pH at the interphase of the papilla matrix and the cellulosic cell wall to induce gel-formation of callose (↑pH). Orange arrows indicate the direction and strength of papilla expansion. Green: plasma membrane, red: cellulose fibrils of the cell wall, blue: callosic papilla matrix and callose fibrils, gray: non-callosic papilla matrix.

These models of delivery and transport processes would be sufficient to explain papillae expansion pointing to the cytosol (Nishimura et al., 2003) and a lateral expansion in the paramural space, which was strongly induced in *Arabidopsis PMR4* overexpression lines where an additional field of callose surrounded the dense core region of the papilla (Ellinger et al., 2013; Naumann et al., 2013). Material or cell wall enzymes, like callose synthases that would be required for a growth of the papilla in these directions, could be delivered along the plasma membrane. However, we recently detected an expansion of the papilla into the pre-existing cellulosic cell wall. Super-resolution microscopy at sites of papillae formation, where we stained callose with aniline blue fluorochrome and the overlying cellulosic cell wall with pontamine fast scarlet 4B, revealed a migration of callose fibrils into the cell wall (Eggert et al., 2014). Only single callose fibrils, which originated from the dense callosic core of the papilla, migrated into and penetrated through the cellulosic cell wall in wild-type *Arabidopsis* leaves. In contrast, a dense network of callose/cellulose fibrils was established along the papilla core region and the lateral field of callose in epidermal leaf cells of *PMR4* overexpression lines at sites of attempted powdery mildew penetration. In addition to this polymer network, a callose layer was formed on top of the cellulosic cell wall (**Figure 1**). We showed that the complex of the callose/cellulose network and the additional callose layer provided enhanced resistance to cell wall degrading enzymes (Eggert et al., 2014), which helped to explain the observed complete penetration resistance to powdery mildew in *PMR4* overexpression plants (Ellinger et al., 2013).

These findings raised the question whether this new direction of papilla expansion would be a regulated process or the consequence of an ongoing callose production at the plasma membrane of the forming papilla. Because the expansion of the callosic papilla into the cellulosic cell wall occurred at sites without contact to the plasma membrane, the previously discussed transport and delivery mechanisms would not apply. Our results from super-resolution microscopy suggest a permeation of callose fibrils through internal cell wall nanopores (Carles and Scallan, 1973; Hubbe et al., 2007). This could be facilitated if callose would have a gel-like condition at these migration sites. In general, a gel-forming property of callose has been described as being pH-dependent (Harada et al., 1968; Saito et al., 1979). Hence, either conditions at the interphase of the callose deposition and the cellulosic cell wall would favor a gel-formation of callose without active regulation or cellular processes might actively regulate pH condition at the interphase. The apoplastic alkalinization that has been discussed as a general stress factor caused by abiotic and biotic stress, would not be sufficient to induce a gel-formation of callose. Even though the apoplastic pH peaked in short term responses to powdery mildew in barley, the apoplastic pH remained acidic (Felle et al., 2004) whereas alkaline condition would be required for gel-formation of callose (Saito et al., 1979). An active, local regulation of the pH might be possible through vesicle-like bodies and MVBs that were detected within the papilla structure (An et al., 2006) and could have access to the callose/cellulose interphase for regulatory activities. A further application of super-resolution microscopy combined with specific and efficient labeling techniques could support the analysis of papilla expansion in all directions.

## Conflict of Interest Statement

The author declares that the research was conducted in the absence of any commercial or financial relationships that could be construed as a potential conflict of interest.
